# Editorial: Microbial ecology and biotechnological potential of alkaline environments

**DOI:** 10.3389/fmicb.2025.1726999

**Published:** 2025-11-04

**Authors:** Anne Postec, Isao Yumoto, Liliana Morales-Barrera, Amare Gessesse, Duncan G. G. McMillan

**Affiliations:** ^1^Aix Marseille Univ, Université de Toulon, CNRS, IRD, MIO, Marseille, France; ^2^Institute for Open and Transdisciplinary Research Initiatives (OTRI), The University of Osaka, Osaka, Japan; ^3^National Polytechnic Institute (IPN), Mexico City, Mexico; ^4^Botswana International University of Science and Technology, Palapye, Botswana; ^5^Department of Biotechnology, Delft University of Technology, Delft, Netherlands; ^6^School of Biological Sciences, University of Reading, Whiteknights, United Kingdom

**Keywords:** alkaliphilic microorganisms, soda lakes, microbial diversity, metabolic adaptation, sulfur disproportionation, ATP synthesis, respiratory chain, biogeography

Alkaliphilic microorganisms can thrive in extraordinarily diverse ecosystems, such as microniches in seemingly ordinary environments, as well as large areas that can be saturated with salts. Indeed, they can exist in ordinary garden soil, indicating the presence of small alkaline niches within conventional environments (Horikoshi, 2004). At the same time, soda lakes representing vast and permanently alkaline ecosystems, characterized by stable high pH and carbonate buffering, and also are home to specialized haloalkaliphilic communities (Sorokin et al., 2014).

Alkaliphiles have evolved unique adaptations that allow them to thrive under conditions of low proton availability, high ionic stress, and limited access to nutrients and trace metals. Despite these physicochemical constraints, alkaline soda lakes rank among the most biologically productive ecosystems on Earth, exhibiting vigorous microbial element cycling and dense phototrophic populations (Haines et al., 2023).

Individual alkaliphilic cells are capable of sustaining entire ecosystems and often exhibit rapid growth. This apparent paradox underscores the extraordinary adaptive potential of alkaliphiles, which have evolved not only to endure harsh conditions, but have harnessed them as key drivers of metabolic innovation. In many cases, environmental constraints become catalytic forces for the development of unique bioenergetic strategies and biosynthetic capabilities.

Because enzymes derived from alkaliphiles exhibit high activity at ambient temperatures and remarkable stability under various conditions, their proteins have long been applied in industrial contexts, such as laundry detergents and food production. Such enzymes include proteases, amylases, and cyclodextrin-producing enzymes (Horikoshi, 2004).

Taken together, these observations suggest that alkaline ecosystems, and the multifactorially adapted microorganisms within them, have emerged as reservoirs of novel enzymes, metabolites, and bioenergetic strategies with promising applications in biotechnology, bioremediation, and sustainable manufacturing.

This Research Topic brings together recent advances in the study of microbial diversity, metabolic innovation, and ecological function in alkaline environments. The contributions span a wide range of approaches, from metagenomics and transcriptomics to culture-based isolation and biochemical characterization offering a multidimensional view of how life persists and flourishes under alkaline environments. Three major themes emerge from this Research Topic, reflecting the multidimensional nature of microbial life in alkaline environments.

## 1 Microbial community structure and ecological resilience

Contributions to this Research Topic highlight how alkaline ecosystems host complex microbial consortia shaped by environmental gradients, dispersal barriers, and biotic interactions.

In Soap Lake (Washington, USA), Vanderlaan et al. combined culture-based and 16S rRNA amplicon sequencing approaches to characterize microbial diversity across oxic and anoxic layers. They isolated over 100 alkaliphilic strains, including cold-adapted *Vibrio*, and identified sulfate-reducing *Deltaproteobacteria* in deep waters. Ren and Wang conducted a global biogeographic survey of soda lake microbiomes using 51 metagenomes from Africa, Asia, and North America. Their analysis revealed strong distance-decay relationships in taxonomic and functional composition, and uneven cross-continent transition rates, highlighting dispersal limitation and regional endemism. Wang et al. investigated the effects of microbial communities during the cultivation of three salt-tolerant plants in severely saline-alkaline soils. Their study showed that plant–microbe interactions can improve soil quality, enhance microbial diversity, and influence pathogen dynamics, with chicory showing the strongest positive impact.

These studies reveal how microbial communities in alkaline environments are shaped by stratification, geographic isolation, and biotic interactions. They highlight ecological complexity and adaptive resilience. Together, they underscore the role of environmental gradients and biological partnerships in structuring microbial diversity and function under alkaline stress.

## 2 Metabolic versatility under alkaline environments

Studies in this Research Topic show that microorganisms thriving in high-pH ecosystems deploy diverse and often unconventional strategies for energy conservation and resource acquisition. In Dziani Dzaha (Mayotte), Duperron et al. used transcriptomic profiling to reveal the stable co-dominance of two phototrophs, *Limnospira platensis* and *Picocystis salinarum*—throughout the water column. Differential gene expression patterns suggested niche partitioning and metabolic flexibility under low-light, low-oxygen conditions. de Jong et al. utilized applied quantitative proteomics to *Caldalkalibacillus thermarum* grown under microaerobic conditions. Their findings revealed oxygen-dependent regulation of terminal oxidases and sodium homeostasis mechanisms, offering insights into respiratory flexibility in alkaliphiles. Sorokin, Merkel, Ziganshin et al. presented growth physiology, genomics, and proteomics of *Desulfurivibrio dismutans* sp. nov., an obligate chemolithoautotroph capable of elemental sulfur disproportionation and nitrate ammonification. Machado et al. explored the genetic and biochemical diversity of terpene biosynthesis in cyanobacterial strains from tropical soda lakes. Their integration of gene mining, synteny analysis, and metabolomics uncovered a rich terpenome, including carotenoids and hopanoids with potential roles in stress tolerance and biotechnological applications. Yumoto proposed a structural hypothesis for ATP production in obligate alkaliphilic *Bacillaceae*. The study describes a membrane-bound H^+^-capacitor formed by cytochrome *c* and membrane-bound DUF2759-domain proteins, enabling efficient proton transfer and high ATP synthesis rates under proton-limited conditions, offering a new perspective on bioenergetics in alkaline environments.

These findings demonstrate the biochemical ingenuity required for alkaliphilic microorganisms to maintain metabolic function in proton-poor, ion-stressed environments, and also demonstrate that microorganisms already adapted to harsh environments have the means to cope with further environmental changes.

## 3 Biotechnological potential and resource recovery

Studies in this Research Topic provide examples of alkaliphilic microorganisms as a rich source of enzymes, metabolites, and bioenergetic strategies with applications in sustainable bioprocesses.

Sorokin, Merkel, Khizhniak et al. described the isolation and characterization of cellulose-mineralizing haloalkaliphilic bacteria from Siberian soda lakes, revealing aerobic and anaerobic consortia with diverse cellulase-producing capabilities. Wen et al. investigated the taxonomic and functional diversity of alkali-tolerant bacteria enriched from the Taklimakan Desert. From five soil samples, they isolated 291 strains spanning 56 genera, with many novel taxa and enzymatic activities including amylase, protease, and cellulase, demonstrating biotechnological potential under extreme conditions. Yi et al. reviewed sustainable phycocyanin production using haloalkaliphilic cyanobacteria. They proposed an integrated bioprocess incorporating direct air capture of CO_2_, microbial consortia for process stability, passive extraction methods, and anaerobic digestion for resource recovery, aligning with circular bioeconomy principles. Wencker et al. developed improved methods for genetic manipulation of the alkaliphile *Halalkalibacterium halodurans*, establishing a markerless genome editing system and rapid transformation protocol. Their work expands the toolkit for studying and engineering extremophilic bacteria.

These studies demonstrate the biotechnological value of alkaliphiles, from cellulose degradation and enzyme production to sustainable pigment synthesis. They introduce new microbial resources, innovative bioprocess designs, and genetic tools for engineering extremophiles. Together, they highlight alkaline microbes as key assets for circular bioeconomy and industrial innovation.

All together, the articles in this Research Topic expand our understanding of microbial life in alkaline environments, from molecular mechanisms and ecological dynamics to applied innovations. They reveal that alkaliphilic microorganisms are not merely survivors of extreme conditions, but active architects of robust and productive ecosystems.

As global interest in sustainable biotechnology and environmental resilience grows, the insights gained from alkaline habitats will be increasingly valuable. Future research may focus on:

- Exploring microbial interactions and co-evolution in stratified or plant-associated alkaline environments.
- Integrating multi-omics approaches to uncover hidden metabolic potential and regulatory networks.
- Elucidating the structural basis of bioenergetic adaptations in proton-limited systems.
- Scaling up bioprocesses that leverage alkaliphilic metabolism for industrial and environmental applications.

We hope this Research Topic inspires further interdisciplinary exploration of alkaline ecosystems and encourages the integration of ecological insight with biotechnological innovation.
